# Drug tolerability and reasons for discontinuation of seven biologics in elderly patients with rheumatoid arthritis -The ANSWER cohort study-

**DOI:** 10.1371/journal.pone.0216624

**Published:** 2019-05-08

**Authors:** Kosuke Ebina, Motomu Hashimoto, Wataru Yamamoto, Toru Hirano, Ryota Hara, Masaki Katayama, Akira Onishi, Koji Nagai, Yonsu Son, Hideki Amuro, Keiichi Yamamoto, Yuichi Maeda, Koichi Murata, Sadao Jinno, Tohru Takeuchi, Makoto Hirao, Atsushi Kumanogoh, Hideki Yoshikawa

**Affiliations:** 1 Department of Orthopaedic Surgery, Osaka University, Graduate School of Medicine, Osaka, Japan; 2 Department of Advanced Medicine for Rheumatic Diseases, Graduate School of Medicine, Kyoto University, Kyoto, Japan; 3 Department of Health Information Management, Kurashiki Sweet Hospital, Kurashiki, Japan; 4 Department of Respiratory Medicine and Clinical Immunology, Osaka University, Graduate School of Medicine, Osaka, Japan; 5 The Center for Rheumatic Diseases, Department of Orthopaedic Surgery, Nara Medical University, Nara, Japan; 6 Department of Rheumatology, Osaka Red Cross Hospital, Osaka, Japan; 7 Department of Rheumatology and Clinical Immunology, Kobe University Graduate School of Medicine, Kobe, Japan; 8 Department of Internal Medicine (IV), Osaka Medical College, Osaka, Japan; 9 First Department of Internal Medicine, Kansai Medical University, Osaka, Japan; 10 Department of Medical Informatics, Wakayama Medical University Hospital, Wakayama, Japan; Keio University, JAPAN

## Abstract

**Background:**

The aim of this study is to evaluate the retention rates and reasons for discontinuation for seven biological disease-modifying antirheumatic drugs (bDMARDs) in a real-world setting of elderly patients (65 years of age or older) with rheumatoid arthritis (RA).

**Methods:**

This multi-center, retrospective study assessed 1,098 treatment courses of 661 patients with bDMARDs from 2009 to 2018 (females, 80.7%; baseline age, 71.7 years; disease duration 10.5 years; rheumatoid factor positivity 81.3%; Disease Activity Score in 28 joints using erythrocyte sedimentation rate, 4.6; concomitant prednisolone dose 2.8 mg/day (45.6%) and methotrexate dose 4.4 mg/week (56.4%); and 60.2% patients were bio-naïve). Treatment courses included abatacept (ABT; n = 272), tocilizumab (TCZ; n = 234), etanercept (ETN; n = 184), golimumab (GLM; n = 159), infliximab (IFX; n = 101), adalimumab (ADA; n = 97), and certolizumab pegol (CZP; n = 51). Drug retention rates and discontinuation reasons were estimated at 36 months using the Kaplan-Meier method and adjusted for potential clinical confounders (age, sex, disease duration, concomitant PSL and MTX, starting date and switched number of bDMARDs) by Cox proportional hazards modeling.

**Results:**

A total of 51.2% of treatment courses were stopped, with 25.1% stopping due to lack of effectiveness, 11.8% due to toxic adverse events, 9.7% due to non-toxic reasons, and 4.6% due to remission. Drug retention rates for each discontinuation reason were as follows; lack of effectiveness [from 55.4% (ETN) to 81.6% (ABT); with significant differences between groups (Cox P<0.001)], toxic adverse events [from 79.3% (IFX) to 95.4% (ABT), Cox P = 0.043], and remission [from 94.2% (TCZ) to 100.0% (CZP), Cox P = 0.58]. Finally, overall retention rates excluding non-toxic reasons and remission for discontinuation ranged from 50.0% (ETN) to 78.1% (ABT) (Cox P<0.001).

**Conclusions:**

ABT showed lowest discontinuation rate by lack of effectiveness and by toxic adverse events, which lead to highest overall retention rates (excluding non-toxic reasons and remission) among seven bDMARDs in adjusted model of elderly RA patients.

## Introduction

Tumor necrosis factor inhibitors (TNFi) were the first biological disease-modifying antirheumatic drugs (bDMARDs) used for rheumatoid arthritis (RA), and evidence has accumulated regarding the safety, effectiveness, and tolerability of adalimumab (ADA), etanercept (ETN), and infliximab (IFX) [1–5]. On the other hand, other TNFi such as golimumab (GLM) (2011) and certolizumab pegol (CZP) (2013) recently received approval in Japan, and the European League against Rheumatism (EULAR) announced a 2013 recommendation regarding the management of RA with bDMARDs, in which CTLA4-Ig [abatacept (ABT)] and anti-interleukin (IL)-6 receptor antibody [tocilizumab (TCZ)], are considered as equivalent as TNFi [6]. However, we still lack reliable evidence which directly compared the safety, effectiveness, and tolerability of these seven bDMARDs.

In patients with RA, the population of older individuals [7], as well as its onset age is rapidly increasing [8]. The treatment strategy of elderly patients is often influenced by its comorbidities (renal impairment, chronic lung disease, et al.) in a real-world setting, although the present treatment recommendation is not distinguished by age or comorbidities [9]. On the other hand, patients with elderly-onset RA is associated with higher inflammation and risk of rapid joint destruction compared to younger-onset RA [10, 11], although elderly RA patients receive less frequent of conventional synthetic DMARDs (csDMARDs) including methotrexate (MTX) and bDMARDs treatment compared with younger RA patients [12].

Thus, investigating the effectiveness and safety of bDMARDs in elderly RA patients is of great interests today. However, randomized controlled trials (RCTs) often recruits younger age of patients with less comorbidities who are different from those in real-world settings [13]. Recently, cohort-based observational studies have increasingly been used to investigate the performance of bDMARDs [1–4, 14–16], and drug retention is considered as an index of safety, effectiveness, and tolerability [4, 17–19]. Treatment selection and discontinuation may be influenced by factors such as differences among attending physicians and patient characteristics, although the national health insurance in our country and multicenter studies may help to decrease these possible bias (bDMARDs can be freely selected by attending physicians’ discretion in our country) [17–19]. And as far as we know, there are no reports comparing the effectiveness and safety of seven bDMARDs in elderly RA patients.

We have recently reported the drug retention and reasons for discontinuation among seven biologics in all age of RA [20], and factors associated with the achievement of bDMARDs-free remission [21] from our cohort. The aim of this multicenter, retrospective study was to clarify the retention rates and reasons for discontinuation of seven bDMARDs in the real-world setting of elderly (65 years of age or older) patients with RA.

## Materials and methods

### Patients

The Kansai Consortium for Well-being of Rheumatic Disease Patients (ANSWER) cohort is an observational multicenter registry of patients with RA in the Kansai district of Japan. Data from patients at seven institutes (Kyoto University, Osaka University, Osaka Medical College, Kansai Medical University, Kobe University, Nara Medial University, and Osaka Red Cross Hospital) were included. From 2009 to 2018, 4,461 patients with RA were registered, and 52,654 serial disease activities were available from the database. Data from patients with RA introducing one of seven bDMARDs (ABT, ADA, CZP, ETN, GLM, IFX, and TCZ; including both intravenous and subcutaneous agents, but excluding bio-similar agents) at 65 years of age or older were retrospectively collected. In this study, patients who fulfilled the 1987 RA classification criteria of the American College of Rheumatology [22], with data on starting and discontinuation dates for bDMARDs, and reasons for discontinuation, were included. In addition, baseline demographic data such as age, sex, disease activity (Disease Activity Score in 28 joints using erythrocyte sedimentation rate [DAS28-ESR]), clinical disease activity index (CDAI), duration of disease, number of previously administered bDMARDs, concomitant doses of methotrexate (MTX) and prednisolone (PSL), rheumatoid factor (RF) and anti-cyclic citrullinated peptide antibody (ACPA) positivity, and Health Assessment Questionnaire [HAQ] disability index [DI] score were also collected [20].

Treatments were administered by the attending rheumatologists in accordance with guidelines of the Japan College of Rheumatology. The starting date of each biologic was classified into three groups (2009–2012, 2013–2015, and 2016–2018). Drug retention was retrospectively evaluated as the duration until definitive treatment interruption. Reasons for discontinuation were analyzed and classified into four major categories: 1) lack of effectiveness (including primary and secondary); 2) disease remission; 3) toxic adverse events (infection, skin or systemic reaction, and other toxic events, including hematologic, pulmonary, renal, cardiovascular complications, and malignancies, etc.); and 4) non-toxic reasons (patient preference, change in hospital, desire for pregnancy, etc.). Physicians were allowed to cite only one reason for discontinuation [20]. The representative facility of this registry is Kyoto University, and this observational study was conducted in accordance with the Declaration of Helsinki, with approval by each ethics committee of seven institutes (Kyoto University, Osaka University, Osaka Medical College, Kansai Medical University, Kobe University, Nara Medial University, and Osaka Red Cross Hospital). This study was approved by the Institutional Ethical Review Board of Osaka University Graduate School of Medicine (approval number: 15300), and the board waived the requirement for patients’ informed consent because of the anonymous nature of the data. Written informed consent was obtained from participants in other institutes.

### Statistical analysis

The survival curves of each biologic explained by specific causes were examined by the Kaplan-Meier method and compared statistically using a stratified log-rank test. The time to discontinuation of biologics was analyzed using multivariate Cox proportional hazards modeling [1]. The proportion of treatment retention rates explained by specific causes was analyzed at 36 months [20], and also adjusted by potential confounders that may influence drug discontinuation and the incidence of adverse events, as previously described (age, sex, disease duration, concomitant PSL and MTX, starting date and switched number of bDMARDs) [1, 14–16, 23]. Statistical analyses were performed using EZR (Saitama Medical Center, Jichi Medical University, Saitama, Japan), a graphical user interface for R (The R Foundation for Statistical Computing, Vienna, Austria) [24]. P<0.05 was considered statistically significant.

## Results

### Baseline characteristics

The study population was selected from all patients with RA in the ANSWER cohort (n = 4461) who fulfilled the inclusion criteria (n = 661; 1098 bDMARDs treatment courses). Baseline demographic and clinical characteristics of patients are shown in Table 1. Overall at baseline, mean age was 71.7 years, 80.7% of participants were female, mean disease duration was 10.5 years, RF positivity was 81.3%, ACPA positivity was 85.5%, mean DAS28-ESR score was 4.6, CDAI was 17.3, and mean HAQ-DI score was 1.3. In addition, mean doses of concomitant medications were prednisolone (PSL) 2.8 mg/day (45.6%) and MTX 4.4 mg/week (56.4%). The bDMARDs being administered for the first agent in 60.2% of treatment courses, for the second agent in 22.1%, and for third or latter agent in 17.7%.

**Table 1:** Clinical characteristics at initiation of each biologic agent.

Variable	ABT (n = 272)	ADA (n = 97)	CZP (n = 51)	ETN (n = 184)	GLM (n = 159)	IFX (n = 101)	TCZ (n = 234)
Age (years)	73.0±6.0	69.9±4.7	73.1±6.8	71.9±5.5	72.5±5.6	69.6±3.2	71.0±5.3
Female sex (%)	79.4	79.4	90.2	81.5	86.2	79.2	76.9
BMI (kg/m^2^)	21.7±3.5	21.9±3.5	22.4±3.6	22.2±3.5	22.8±3.5	22.6±3.4	22.0±3.3
Disease duration (years)	10.6±11.2	10.1±11.2	9.1±10.5	10.5±10.2	11.1±11.5	9.1±11.3	11.0±10.9
RF positivity (%)	83.4	79.0	89.4	85.2	73.7	75.3	82.8
ACPA positivity (%)	89.9	82.7	85.1	86.2	82.6	81.0	85.6
DAS28-ESR	4.5±1.2	4.5±1.2	5.1±1.5	4.7±1.3	4.4±1.3	5.0±1.5	4.8±1.3
CDAI	16.9±9.7	15.7±9.0	21.8±12.7	17.6±10.0	16.1±10.7	21.3±14.0	16.8±9.8
HAQ-DI	1.2±0.8	1.0±0.7	1.7±0.9	1.2±0.8	1.3±0.8	1.3±1.0	1.3±0.9
PSL usage (%)	46.8	41.1	37.3	47.8	41.4	48.4	47.8
PSL dose (mg/day)	3.1±7.3	2.9±4.9	1.7±2.6	2.8±3.6	2.3±3.6	3.1±5.9	2.8±3.9
MTX usage (%)	46.1	74.7	52.9	44.9	66.9	100.0	46.1
MTX dose (mg/week)	3.4±4.2	6.1±4.2	4.2±4.6	3.5±4.3	5.2±4.4	8.2±2.5	3.6±4.3
Starting date (2009–2012) (%)	16.1	54.6	0	56.5	17.0	67.3	30.8
Starting date (2013–2015) (%)	65.1	39.2	70.6	37.5	50.9	29.7	52.1
Starting date (2016–2018) (%)	18.8	6.2	29.4	6.0	32.1	3.0	17.1
1^st^ bio (%)	66.5	59.8	58.8	71.2	45.3	86.1	43.6
2^nd^ bio (%)	18.0	29.9	5.9	17.9	30.2	10.9	29.9
≥3rd bio (%)	15.5	10.3	35.3	10.9	24.5	3.0	26.5

Values represent mean ± standard deviation (SD), unless otherwise noted. ABT = abatacept, ADA = adalimumab, CZP = certolizumab pegol, ETN = etanercept, GLM = golimumab, IFX = infliximab, TCZ = tocilizumab, BMI = body mass index, RF = rheumatoid factor, ACPA = anti-cyclic citrullinated peptide antibody, DAS28-ESR = Disease Activity Score in 28 joints using erythrocyte sedimentation rate, CDAI = clinical disease activity index, HAQ-DI = Health Assessment Questionnaire disability index, PSL = prednisolone, MTX = methotrexate, bio = biologic agent.

### Drug retention

Overall, 562 treatment courses (51.2%) were stopped by 36 months. A total of 275 treatment courses (25.1%) were stopped due to lack of effectiveness, 130 treatment courses (11.8%) due to toxic adverse events, 106 treatment courses (9.7%) due to non-toxic reasons, and 51 treatment courses (4.6%) due to remission.

### Causes for discontinuation

**Fig 1:**
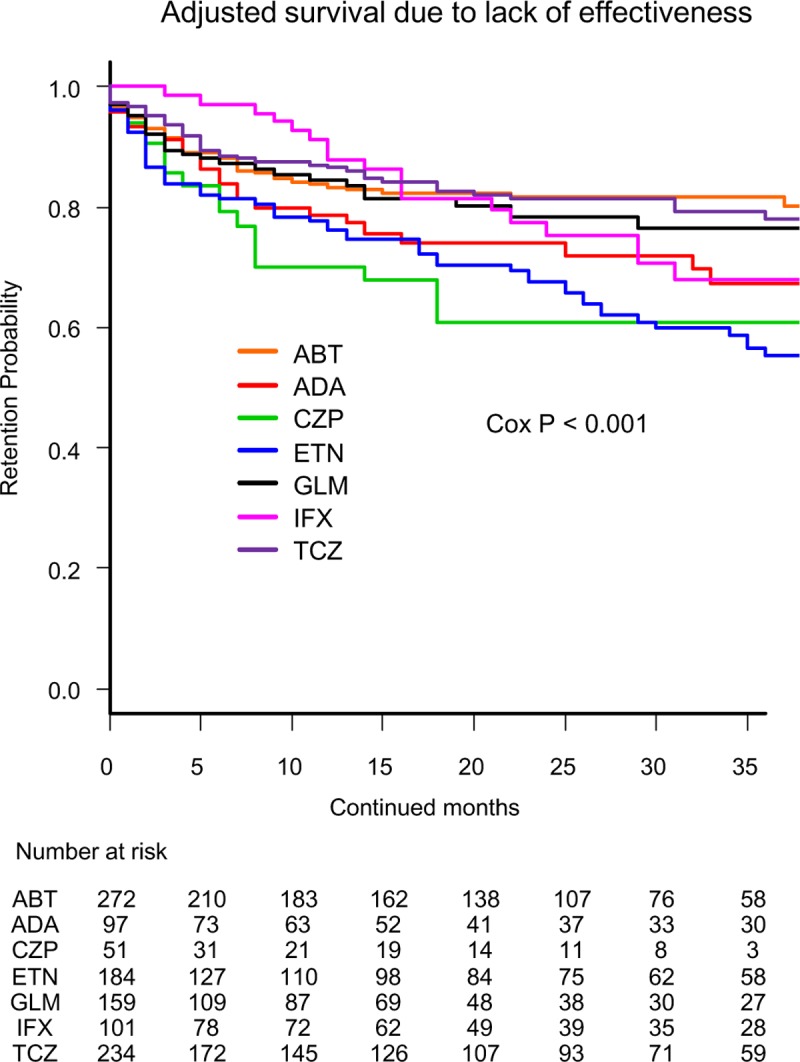
Drug survival rates due to lack of effectiveness in adjusted cases. Adjusted confounders were baseline age, sex, disease duration, concomitant PSL and MTX, starting date and switched number of bDMARDs. ABT = abatacept, ADA = adalimumab, CZP = certolizumab pegol, ETN = etanercept, GLM = golimumab, IFX = infliximab, TCZ = tocilizumab, bDMARDs = biological disease-modifying antirheumatic drugs.

**Fig 2:**
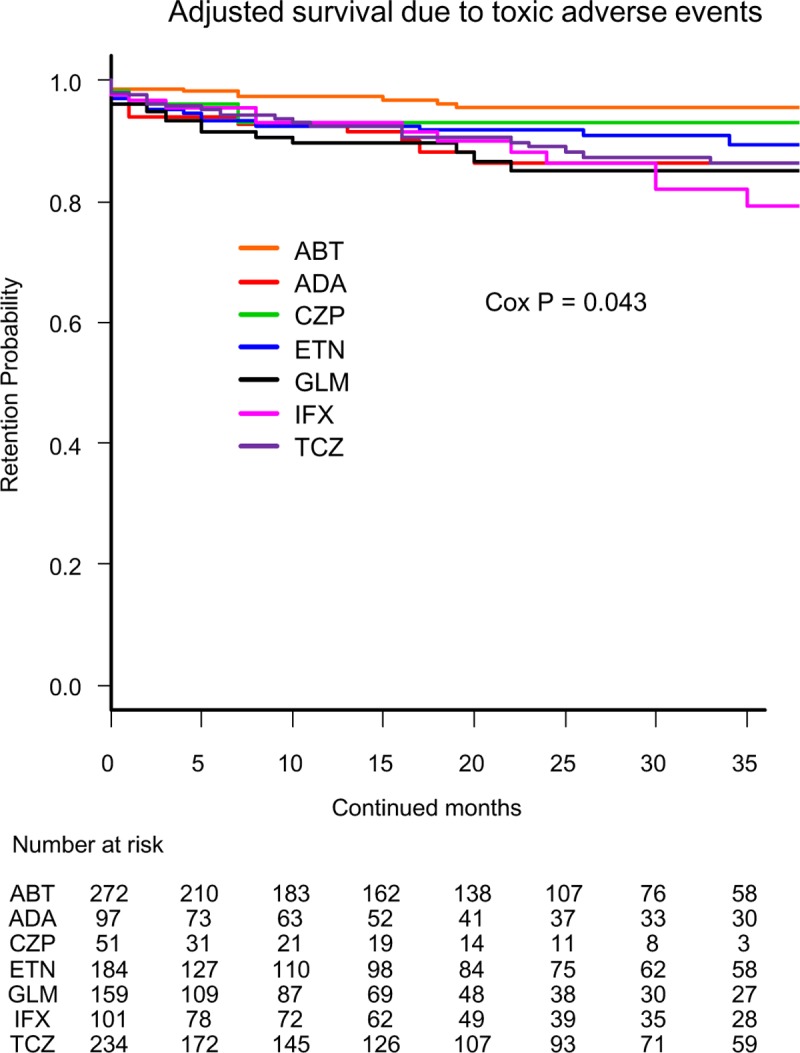
Drug survival rates due to toxic adverse events in adjusted cases. Adjusted confounders were baseline age, sex, disease duration, concomitant PSL and MTX, starting date and switched number of bDMARDs. ABT = abatacept, ADA = adalimumab, CZP = certolizumab pegol, ETN = etanercept, GLM = golimumab, IFX = infliximab, TCZ = tocilizumab, bDMARDs = biological disease-modifying antirheumatic drugs.

**Fig 3:**
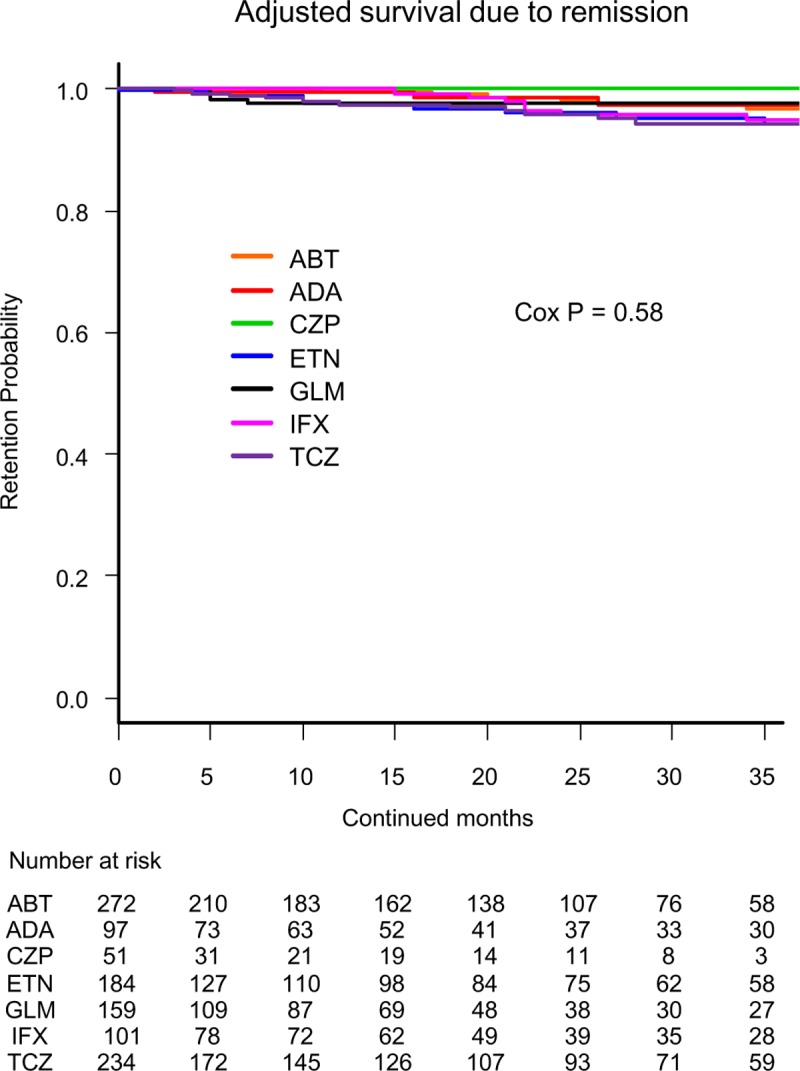
Drug survival rates due to remission in adjusted cases. Adjusted confounders were baseline age, sex, disease duration, concomitant PSL and MTX, starting date and switched number of bDMARDs. ABT = abatacept, ADA = adalimumab, CZP = certolizumab pegol, ETN = etanercept, GLM = golimumab, IFX = infliximab, TCZ = tocilizumab, bDMARDs = biological disease-modifying antirheumatic drugs.

**Fig 4:**
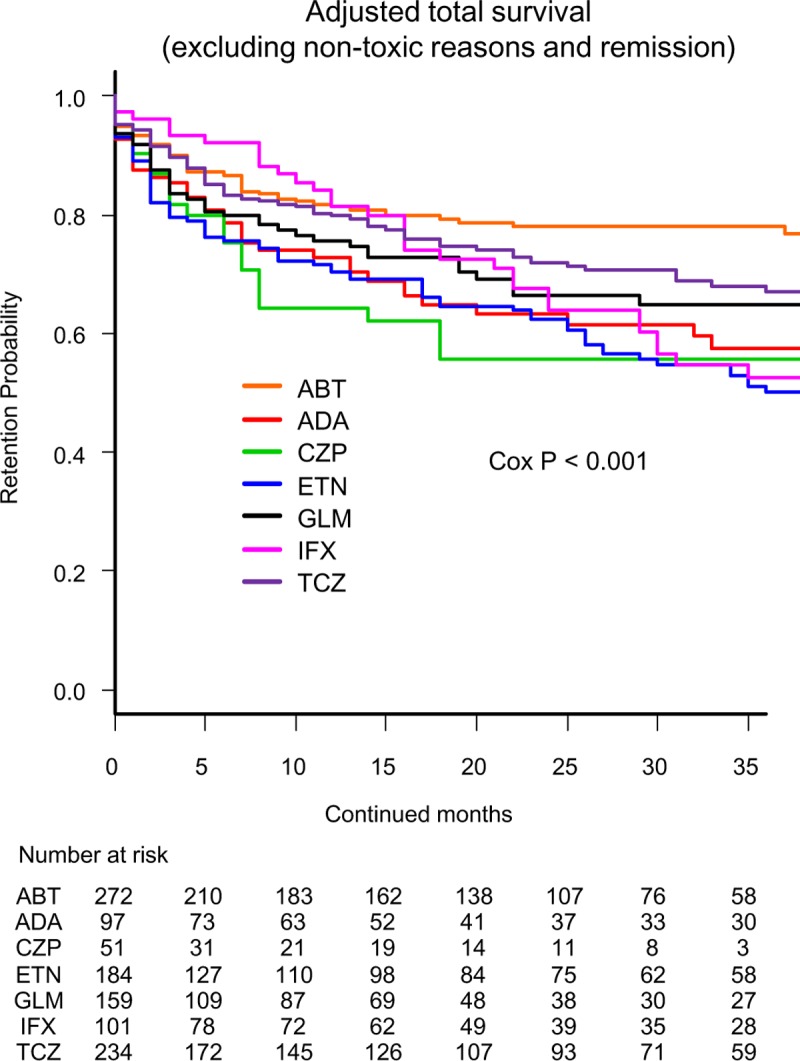
Overall drug survival rates (excluding non-toxic reasons and remission) in adjusted cases. Adjusted confounders were baseline age, sex, disease duration, concomitant PSL and MTX, starting date and switched number of bDMARDs. ABT = abatacept, ADA = adalimumab, CZP = certolizumab pegol, ETN = etanercept, GLM = golimumab, IFX = infliximab, TCZ = tocilizumab, bDMARDs = biological disease-modifying antirheumatic drugs.

Cause-specific cumulative discontinuation rates were assessed using Kaplan-Meier estimates in adjusted models for cofounders using Cox proportional hazards regression modeling (Figs 1–4). At 36 months, drug retention rates due to lack of effectiveness (Fig 1) were as follows: ABT (81.6%), TCZ (78.0%), GLM (76.5%), IFX (68.0%), ADA (67.3%), CZP (60.9%), and ETN (55.4%) (Cox P<0.001). Drug retention rates due to toxic adverse events (Fig 2) were as follows: ABT (95.4%), CZP (92.9%), ETN (89.5%), ADA (86.3%), TCZ (86.3%), GLM (85.0%), and IFX (79.3%) (Cox P = 0.043).

Drug retention rates due to remission (Fig 3) were as follows: CZP (100.0%), GLM (97.7%), ADA (97.5%), ABT (96.8%), IFX (94.8%), ETN (94.4%), and TCZ (94.2%) (Cox P = 0.58).

Total drug retention rates (excluding non-toxic reasons and remission) were analyzed using Kaplan-Meier estimates in adjusted model using Cox proportional hazards regression modeling (Fig 4). At 36 months, drug retention rates were as follows: ABT (78.1%), TCZ (66.8%), GLM (64.8%), ADA (57.6%), CZP (55.6%), IFX (52.5%), and ETN (50.0%), (Cox P<0.001).

Hazard ratios (HRs) and 95% confidence intervals (CI) for discontinuation due to each specific cause were calculated using multivariate Cox proportional hazards modeling, adjusted for age, sex, disease duration, concomitant PSL and MTX, starting date and switched number of bDMARDs (Table 2). HRs for total discontinuation (excluding non-toxic reasons and remission) were significantly higher with ADA (HR = 1.90, P = 0.0019), CZP (HR = 2.17, P = 0.0025), ETN (HR = 2.36, P<0.001), and IFX (HR = 1.90, P = 0.0021) compared with ABT, and differences were significant between the seven bDMARDs (P<0.001). In terms of HRs for discontinuation due to lack of effectiveness, CZP (HR = 2.17, P = 0.0056) and ETN (HR = 2.22, P<0.001) showed significantly higher rates compared with ABT. Differences were significant between the seven bDMARDs (P<0.001).

**Table 2:** Causes of treatment discontinuation at 36 months (Cox proportional hazards model, adjusted by baseline age, sex, disease duration, concomitant PSL and MTX, starting date and switched number of bDMARDs).

	Reference	HR (95% CI)						
Variable	ABT (n = 272)	ADA (n = 97)	CZP (n = 51)	ETN (n = 184)	GLM (n = 159)	IFX (n = 101)	TCZ (n = 234)	P- value
Total discontinuation (excluding non-toxic reasons and remission)	1	1.90 (1.27–2.85)**	2.17 (1.31–3.59) **	2.36 (1.70–3.30)***	1.46 (0.99–2.15)	1.90 (1.26–2.87)**	1.30 (0.91–1.85)	<0.001
Lack of effectiveness	1	1.53 (0.96–2.47)	2.17 (1.25–3.74)**	2.22 (1.53–3.20)***	1.09 (0.69–1.73)	1.37 (0.83–2.27)	0.96 (0.64–1.45)	<0.001
All toxic adverse events	1	3.16 (1.36–7.35)**	2.23 (0.61–8.15)	2.50 (1.15–5.43)**	3.58 (1.63–7.82)**	3.62 (1.58–8.26)**	3.04 (1.45–6.38)**	0.043
Non-toxic reasons	1	1.83 (0.82–4.09)	1.13 (0.33–3.85)	1.19 (0.57–2.48)	1.39 (0.66–2.93)	2.22 (0.96–5.16)	1.23 (0.63–2.38)	0.62
Remission	1	1.14 (0.33–4.02)	<0.001 (0.00-infinite)	2.14 (0.79–5.75)	1.61 (0.45–5.71)	1.87 (0.66–5.34)	2.48 (0.97–6.34)	0.58

HR = hazard ratio; 95% CI = 95% confidence interval, ABT = abatacept, ADA = adalimumab, CZP = certolizumab pegol, ETN = etanercept, GLM = golimumab, IFX = infliximab, TCZ = tocilizumab. Differences between drugs were assessed using the Cox-P value.

* P<0.05,

**P<0.01,

*** P<0.001.

In terms of HRs for discontinuation due to all toxic adverse events, ADA (HR = 3.16, P = 0.0076), ETN (HR = 2.50, P = 0.0020), GLM (HR = 3.58, P = 0.0014), IFX (HR = 3.62, P = 0.0023), and TCZ (HR = 3.04, P = 0.0032) showed a significantly higher rate compared with ABT, and the difference was significant between the seven bDMARDs (P = 0.043).

On the other hand, no significant differences were observed in HRs for discontinuation due to non-toxic reasons (P = 0.62) or remission (P = 0.58).

## Discussion

This retrospective study was designed to evaluated the retention rates and reasons for discontinuation for seven bDMARDs in a real-world setting of elderly (65 years of age or more) patients with RA.

As for the effectiveness of bDMARDs in elderly patients, ABT was well tolerated and similarly efficacious in both of the non-elderly (<65 years) and elderly (≥65 years) RA patients [25]. In addition, from the post hoc analysis of post-marketing surveillance in Japan, GLM showed comparable improvement of disease activity between younger (<75 years) and elderly (≥75 years) patients [26].

In terms of toxic adverse events, ABT showed a lower risk of hospitalized infection compared with TNFi [27], and also all other bDMARDs [28]. Another large cohort study demonstrated that ABT was associated with a 20% reduced risk of cardio-vascular events versus TNFi [29], and another recent report suggested the effectiveness of ABT in RA-associated interstitial lung disease [30], which may lead to lower toxic adverse events of ABT in the present study. On the other hand, recent report showed that the risk for toxic adverse events such as lupus-like events and vasculitis-like events tended to be lowest with CZP compared with other TNFi [31]. In addition, the incidence of serious infections across bDMARDs in patients with RA was not higher with CZP compared with other bDMARDs [32].

Finally, with respect to the total drug persistency, Jones et al. showed that treatment persistence was longer on ABT or TCZ followed by TNFi [33], and we have also reported that both ABT and TCZ showed higher retention rate compared with other TNFi in all ages [20]. Concerning patients with TNFi failure, both ABT and TCZ showed similar substantial improvement in clinical disease activity [34], and also good retention rates [35]. Concerning TNFi, a recent report demonstrated that GLM showed higher persistency compared with other TNFi when matched with propensity score in Japanese RA patients [36]. Taken together, ABT, TCZ, and GLM may have some advantages in total drug persistency compared to other bDMARDs, which was consistent with the present study. This phenomenon may be partially due to small dose and ratio of concomitant MTX in this study, which may affect other TNFi effectiveness more stronger than that of non-TNFi.

Other factors affecting bDMARDs retention and response have been reported. Higher age [3], sex [5], concomitant PSL [3], high DAS28 or HAQ [3, 15, 37], absence or low dose of combined MTX [3, 15], and the number of previously used bDMARDs [15] were the negative predictors in previous studies. With reference to these previous reports, we selected age, sex, disease duration, concomitant PSL and MTX, starting date and switched number of bDMARDs as the adjustment confounders in the present study.

Regarding the efficacy of low-dose MTX in Japanese populations compared with western populations, intraerythrocyte MTX-polyglutamate (MTX-PG) concentrations, which have been suggested to be a useful biomarker of efficacy, reached 94 nmol/L with 10.3 mg/week of MTX in Japanese, compared to 65 nmol/L with 13.4 mg/week of MTX in the United States [38]. As a result, a relatively low dose of MTX may exhibit positive effects on bDMARD retention in Japanese populations compared with western populations.

Some limitations to this study need to be considered. First, the judgment and reasons for discontinuation (such as lack of effectiveness or remission) depended on the decisions of each physician, without standardized criteria. Second, the backgrounds of patients differed between the agents, which may affect the results even adjusted by potent cofounders, and comorbidities could not be evaluated. Third, the minor dose changes of bDMARDs, MTX, and PSL could not be monitored. Fourth, the difference of intravenous and subcutaneous bDMARDs, and the presence of other csDMARDs could not be determined. Fifth, CZP was licensed most recently (2013) among seven bDMARDs in our country, which may lead to small number of prescription that may affect the results.

However, the strength of this study is that this is the first study comparing treatment persistency and discontinuation reasons of seven bDMARDs in elderly RA patients, and also the treatment choice and discontinuation judgments were based on a real-world setting.

## Conclusions

ABT showed lowest discontinuation rate by lack of effectiveness and by toxic adverse events, which lead to highest overall retention rates (excluding non-toxic reasons and remission) among seven bDMARDs in adjusted model of elderly RA patients.
